# Hyperbaric Treatment Stimulates Chaperone-Mediated Macroautophagy and Autophagy in the Liver Cells of Healthy Female Rats

**DOI:** 10.3390/ijms251910476

**Published:** 2024-09-28

**Authors:** Agnieszka Pedrycz, Mariusz Kozakiewicz, Mansur Rahnama, Marek Kos, Ewelina Grywalska, Marietta Bracha, Anna Grzywacz, Iwona Bojar

**Affiliations:** 1 Faculty of Medicine and Health Sciences, University of Applied Sciences in Tarnow, Mickiewicza 8, 33-100 Tarnów, Poland; apw4@wp.pl; 2 Ludwik Rydygier Collegium Medicum in Bydgoszcz, Nicolaus Copernicus University in Toruń, Jagiellońska 13/15, 85-067 Bydgoszcz, Poland; markoz@cm.umk.pl; 3 Departament of Dental Surgery, Medical University of Lublin, Chodźki 6, 20-093 Lublin, Poland; mansur.rahnama@umlub.pl; 4 Department of Public Health, Medical University of Lublin, Chodźki 1, 20-093 Lublin, Poland; marek.kos@umlub.pl; 5 Department of Experimental Immunology, Medical University of Lublin, Chodźki 4a, 20-093 Lublin, Poland; 6 Department of Geriatrics, Faculty of Health Sciences, Nicolaus Copernicus University in Toruń, Jagiellońska 15, 85-067 Bydgoszcz, Poland; marietta.bracha@cm.umk.pl; 7 Independent Laboratory of Behaviour Genetics and Epigenetics, Pomeranian Medical University in Szczecin, Rybacka 1, 70-204 Szczecin, Poland; anna.grzywacz@pum.edu.pl; 8 Department of Women’s Health, Institute of Rural Health, 20-093 Lublin, Poland; iwonabojar75@gmail.com

**Keywords:** hyperbaric treatment, autophagy, viruses, chaperones (HSC73), liver

## Abstract

The role of autophagy goes far beyond the elimination of damaged cellular components and the quality control of proteins. It also cleanses cells from inclusions, including pathogenic viruses, and provides energy-forming components. The liver, which is an organ with increased metabolism, is made up of cells that are particularly vulnerable to damage. Therefore, detoxification of liver cells in the process of autophagy has become a very important issue clinically. The aim of this study was an immunohistochemical evaluation of proteins activated in rat liver cells at different stages of hyperbaric autophagy. The rats used for the study were randomly divided into six equivalent groups—three control groups and three experimental groups. Animals from the experimental groups were subjected to hyperbaric treatment in a hyperbaric chamber, with a pressure of 1.6 ATA for 120 min. They breathed atmospheric air. Rats were decapitated within 5 or 10 days after removal from the chamber. Immunohistochemical reactions with beclin 1, LC3B, RAB7, and HSC73 proteins were carried out on preparations made from liver slices. A three-step labeled streptavidin–biotin detection method of paraffin blocks (LSAB three-step) was used for immunohistochemical research. The results were evaluated using computer programs for morphometric analysis of microscopic images by calculating the mean surface areas occupied by a positive immunohistochemical reaction in individual groups for all antibodies tested. Increased closure of substrates in the autophagosome (beclin 1) induced late endosome transport and accelerated autophagosome maturation process (RAB7). Furthermore, a larger number of autophagosomes (LC3B) was observed in liver cells immediately after the cessation of hyperbaric activity; however, this decreased after 5 days. During this time, chaperone-mediated autophagy (HSC73) was observed on a larger scale. This means that increased macroautophagy induced by hyperbaric treatment weakens with time that has elapsed since the cessation of high pressure, whereas similarly induced chaperone-mediated autophagy intensifies over time.

## 1. Introduction

Autophagy is a highly conserved evolutionary process found in all eukaryotic cells, from single-celled yeast to multicellular organisms like mammals. This catabolic process primarily functions as an intracellular degradation system of the large-molecule components of the cytoplasm, especially long-life proteins and entire organelles [1,2,3]. Stimulated autophagy clears cells of protein aggregates, sugars, fats, viruses, and other pathogenic microorganisms that damage the cell [4,5,6]. There are three primary forms of autophagy depending on how the substrate is delivered to lysosomes: macroautophagy, microautophagy, and chaperone-mediated autophagy (CMA).

Macroautophagy is the most common form of autophagy. During its course, a fragment of the cytoplasm is surrounded by the formation of a C-shaped double membrane called a phagophore [7]. Both ends of the phagophore lengthen, closing inside part of the cytoplasm along with proteins or whole organelles. The 300–900 nm bubble formed in this way, called the autophagosome, undergoes a maturation process. In the process of maturation, autophagosomes with lysosomes merge, which leads to the formation of autophagolysosomes. The process that takes place in these structures is a process of degradation of large-molecule substrates to their basic components—amino acids, simple sugars and fatty acids—with the use of lysosomal hydrolytic enzymes. Macroautophagy is the main source of amino acids and other basic ingredients needed by the body in conditions of lack of food [8]. Key proteins involved in macroautophagy include beclin 1, which initiates the process, LC3B, a marker for autophagosome maturation, and RAB7, which regulates transport from early to late endosomes and lysosomes.

Microautophagy is the least understood form of autophagy. During this process, a fragment of the cytoplasm with soluble proteins or organelles is surrounded by a lysosomal membrane, and through endocytosis penetrates the interior of the lysosomes [8,9].

During CMA, selected cytosolic proteins are transported into lysosomes through specific receptors on their membrane. The receptor recognizes and binds the chaperone–substrate complex, and the second chaperone protein, which is present inside the lysosome, allows the substrate to be translocated [8,10,11].

The liver plays a crucial role in the metabolism of proteins, fats, carbohydrates, and detoxification processes. It was through studies on hepatocytes that lysosomes were identified, leading to the discovery of autophagy [10,11]. Therefore, for this experiment, the authors focused their search on factors that stimulate autophagy in the liver. Liver cells are among the first ones to respond to stress by stimulating autophagy. Similarly, neurons, pulmonary alveoli, and kidney cells quickly respond with autophagy. Intensive studies of the autophagy process in liver cells in recent years have shown that lysosome-modulated degradation is important not only for maintaining liver homeostasis under normal physiological conditions but also for the appropriate response of this organ to stressors such as toxic proteins, metabolic dysregulation, infections, and tumorigenesis [11,12].

Stimulated autophagy is a desirable process that can cleanse cells of damaging factors in their area. Several methods to induce autophagy have been explored, often based on the induction of cellular stress such as hypoxia, infection, or oxidative stress, which have considerable side effects [13]. Hyperbaric treatment has not been described as a factor that can stimulate autophagy.

This study aimed to explore whether elevated pressure (1.6 ATA = 16 m underwater depth) using air as a breathing medium can trigger autophagy. Previous research suggests that autophagy plays a role in combating infections [14,15,16]. This study examined whether hyperbaric treatment, a minimally invasive method that that may help eliminate COVID-19 genetic material from cells, can trigger autophagy. Exposure to a depth of 16 m can be achieved in hyperbaric chambers without the need for diving. The accessibility of this method offers potential for widespread use in treating COVID-19 patients.

The aim of this study was to evaluate immunohistochemical expression of proteins activated in rat liver cells at different stages and in various types of autophagy induced by pressure higher than atmospheric pressure (1.6 ATA for 120 min). The proteins assessed were beclin 1, which initiates macroautophagy, LC3B, a marker of macroautophagy, RAB7, involved in transport from early to late endosomes and lysosomes, and HSC73, which is involved in chaperone-mediated autophagy.

## 2. Results

### 2.1. Baseline Characteristics

No significant differences were noted in the macroscopic image taken for liver tests of female rats from the control and experimental groups. The structure of the organ did not deviate from the textbook norm.

In all groups, histological images of the liver on microscopy were also similar. It was an image of a normal organ without pathological changes. The spaces between the stromal elements and vessels were filled by hepatocytes arranged in liver lobules. Hepatocytes contained one or two nuclei. In the central part of the hepatic lobules, cross sections were visible through the middle veins, the walls of which were characterized by the correct structure, and endothelial cells were visible. In the corners of the three- to six-angled lobules, gate-bile spaces (portal spaces) filled with connective tissue with cross sections through arteries, veins, and interlobular bile ducts were visible. The next visible cells were liver macrophages present in the sinusoidal endothelial cells, i.e., Kupffer–Browicz cells.

### 2.2. Reaction for Beclin 1 Protein

**Figure 1:**
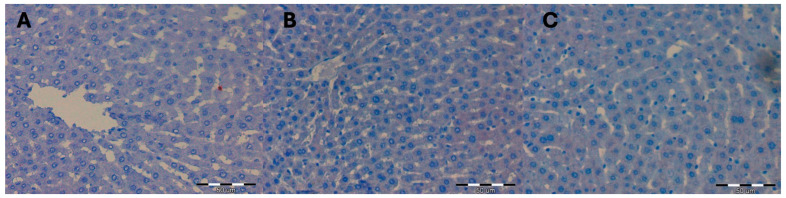
(**A**) KI control group. Invisible immunohistochemical reaction for beclin 1 in the liver fragment of a female rat that was decapitated on the 1st day of the experiment. H + AEC dyeing. (**B**) KII control group. Invisible immunohistochemical reaction for beclin 1 in a fragment of the liver of a female rat that was decapitated on the 5th day of the experiment. Dyeing H + AEC. (**C**) KIII control group. Invisible immunohistochemical reaction for beclin 1 in a fragment of the liver of a female rat that was decapitated on the 10th day of the experiment. Dyeing H + AEC.

There was no difference between the groups on assessment of the mean surface areas of liver preparations occupied by a positive immunohistochemical reaction for beclin 1 in the livers of female rats from control groups, i.e., not subjected to hyperbaric treatment. The color reaction for beclin 1 was not visible on any of the observed liver preparations (Figure 1) (Table 1).

**Figure 2:**
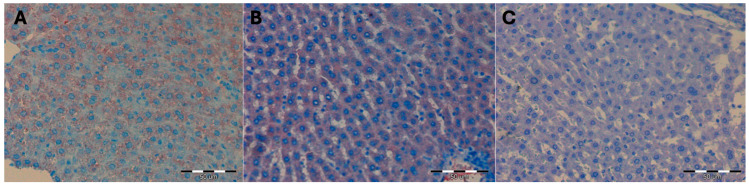
(**A**) DHI experimental group. Moderately severe immunohistochemical reaction for beclin 1 in a fragment of the liver of a female rat that underwent hyperbaric treatment and was decapitated immediately after removal from the hyperbaric chamber. H+AEC dyeing. (**B**) Experimental group DHII. Moderately severe immunohistochemical reaction for beclin 1 in a fragment of the liver of a female rat that underwent hyperbaric treatment and was decapitated 5 days after removal from the hyperbaric chamber. H + AEC dyeing. (**C**) Experimental group DHIII. Absent immunohistochemical reaction for beclin 1 in a liver fragment of a female rat that underwent hyperbaric treatment and was decapitated 10 days after removal from the hyperbaric chamber. Dyeing H + AEC.

The mean surface area occupied by a positive immunohistochemical reaction for beclin 1 in the liver of female Wistar rats subjected to high pressure and decapitated immediately after removal from the hyperbaric chamber (DHI, mean 1005, 35 μm^2^ +/− 233.21) increased statistically significantly compared to the control groups (*p <* 0.001). It remained at the same level (mean 1100.34 μm^2^ +/− 212, 45; *p* = 0.83) in the group of female rats decapitated 5 days after removal from the hyperbaric chamber (DHII), whereas in the group of rats decapitated 10 days after removal from the hyperbaric chamber (DHIII), it was decreased (mean 290, 35 μm^2^ +/− 88, 32) statistically significantly (*p* < 0.001) (Figure 2) (Table 2 and Table 3).

Hyperbaric treatment stimulated the process of closing the substrate in the autophagosome, i.e., covering it with a phagophore and finally creating an autophagosome during autophagy in rat liver cells, which was observed in animals decapitated immediately after removal from the hyperbaric chamber. This phenomenon persisted 5 days after the cessation of high pressure. Ten days after the animals were removed from the hyperbaric chamber, the process of covering it with a phagophore of the substrate and the formation of the autophagosome was no longer observed (no reaction for beclin 1).

### 2.3. Reaction for LC3B Protein

**Figure 3:**
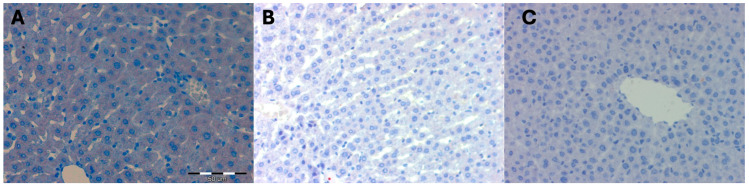
(**A**) Control group KI. Invisible immunohistochemical reaction for LC3B in a fragment of the liver of a female rat that was decapitated on 1st day of the experiment. H + AEC staining. (**B**) KII control group. Invisible immunohistochemical reaction for LC3B in a fragment of the liver of a female rat that was decapitated on the 5th day of the experiment. Magnification approx. 180×. Dyeing H + AEC. (**C**) KIII control group. Invisible immunohistochemical reaction for LC3B in a fragment of the liver of a female rat that was decapitated on the 10th day of the experiment. Magnification approx. 180×. H + AEC staining.

No statistically significant differences were observed in the mean area occupied by a positive immunohistochemical reactions for LC3B in the livers of female rats from control groups not subjected to hyperbaric treatment and decapitated on the 5th day of the KII experiment (mean 350.00 +/− 6.10) or the 10th day of the KIII experiment (mean 350.00 +/− 8.04; *p* = 1). The mean area of the area affected by a positive immunohistochemical reaction for LC3B in the liver of female control rats studied on day 1 of the experiment was statistically significantly smaller in KI (mean 302.33 +/− 8.74; *p* = 0.001 and *p* = 0.002) than that recorded in the livers of female rats from control groups: KII, decapitated on the 5th day (mean 350.00 +/− 6.10), and KIII, decapitated on the 10th day of the experiment (Wed. 350.00 +/− 8.04). (Figure 3) (Table 4).

**Figure 4:**
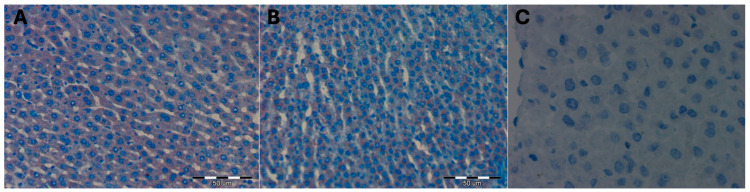
(**A**) Experimental group DHI. Moderately severe immunohistochemical reaction for LC3B in a liver fragment of a female rat that underwent hyperbaric treatment and was decapitated immediately after removal from the hyperbaric chamber. H+AEC dyeing. (**B**) Experimental group DHII. Moderately severe immunohistochemical reaction for LC3B in a liver fragment of a female rat that underwent hyperbaric treatment and was decapitated 5 days after removal from the hyperbaric chamber. Dyeing H + AEC. (**C**) Experimental group DHIII. Absent immunohistochemical reaction for LC3B in a liver fragment of a female rat that underwent hyperbaric treatment and was decapitated 10 days after removal from the hyperbaric chamber. Magnification approx. 360×. H + AEC staining.

The mean surface area occupied by a positive immunohistochemical reaction for LC3B in the liver of female Wistar rats subjected to high pressure and decapitated immediately after removal from the DHI hyperbaric chamber (mean 1042.67 +/− 367.86) increased significantly statistically compared to the control groups (*p* < 0.001). It remained at the same level (mean 1038.33 +/− 294.38; *p* = 0.99) in the group of DHII female rats decapitated 5 days after removal from the hyperbaric chamber, and in the group of female rats DHIII decapitated after 10 days after removal from the hyperbaric chamber, it decreased (mean 305.00 +/− 42.72) statistically significantly (*p* = 0.01) (Figure 4) (Table 5 and Table 6).

Hyperbaric treatment stimulated the process of autophagosome formation during autophagy (of which the only reliable marker is currently the LC3B protein) in rat liver cells, which was observed in animals decapitated immediately after removal from the hyperbaric chamber. This phenomenon persisted 5 days after the cessation of high pressure. On the 10th day after the removal of animals from the hyperbaric chamber, the process of autophagosome formation during autophagy in rat liver cells was no longer observed, which was confirmed by the lack of reaction for LC3B.

### 2.4. Reaction for the RAB7 Protein

**Figure 5:**
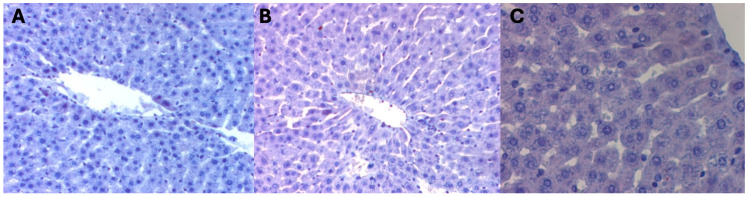
(**A**) Control group KI. Invisible immunohistochemical reaction for RAB7 in the liver fragment of a female rat that was decapitated on the 1st day of the experiment. Magnification approx. 300×. H + AEC staining. (**B**) KII control group. Invisible immunohistochemical reaction for RAB7 in a fragment of the liver of a female rat that was decapitated on the 5th day of the experiment. Magnification approx. 300×. H+AEC staining. (**C**) KIII control group. Invisible immunohistochemical reaction for RAB7 in a fragment of the liver of a female rat that was decapitated on the 10th day of the experiment. Magnification approx. 500×. H + AEC staining.

No statistically significant differences were observed in the mean area occupied by a positive immunohistochemical reaction for the RAB7 protein in the livers of female rats from control groups and thus not subjected to hyperbaric treatment. The color reaction was not visible on any of the observed liver preparations (Table 7) (Figure 5).

**Figure 6:**
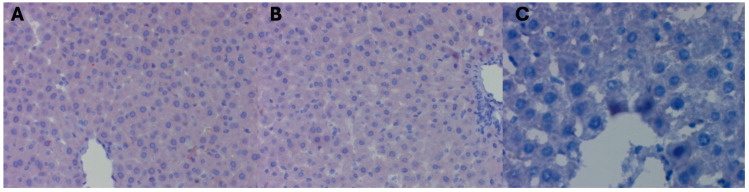
(**A**) Experimental group DHI. Moderately severe immunohistochemical reaction for RAB7 in a fragment of the liver of a female rat that underwent hyperbaric treatment and was decapitated immediately after removal from the hyperbaric chamber. Magnification approx. 300×. H + AEC staining. (**B**) Experimental group DHII. Moderately severe immunohistochemical reaction for RAB7 in a fragment of the liver of a female rat that underwent hyperbaric treatment and was decapitated 5 days after removal from the hyperbaric chamber. Magnification approx. 300×. H + AEC staining. (**C**) Experimental group DHIII. Absent immunohistochemical reaction for RAB7 in a liver fragment of a female rat that underwent hyperbaric treatment and was decapitated 10 days after removal from the hyperbaric chamber. Magnification approx. 500×. H + AEC staining.

The mean surface area occupied by a positive immunohistochemical reaction for the RAB7 protein in the liver of females of the Wistar rat strain subjected to high pressure and decapitated immediately after removal from the hyperbaric chamber DHI (mean 1006.67 +/− 90.18) increased statistically significantly compared to the control groups (*p* = 0.001). It remained at the same level (mean 1030.33 +/− 157.16) in the group of females in DHII decapitated after 5 days after removal from the hyperbaric chamber (*p* = 0.83). In the group of females in DHIII decapitated after 10 days after removal from the hyperbaric chamber, it statistically significantly decreased (mean 296.66 +/− 40.41; *p* = 0.001) when compared to the DHI and DHII groups and the values represented by the control groups (Table 8 and Table 9; Figure 6).

Hyperbaric treatment stimulated the process of late endosome transport and the process of autophagosome maturation during autophagy in rat liver cells, which was observed in animals decapitated immediately after removal from the hyperbaric chamber. This phenomenon persisted for up to 5 days after the cessation of high pressure. On the 10th day after the removal of the animals from the hyperbaric chamber, the process of autophagosome maturation and late endosome transport during autophagy in the liver cells of female rats was no longer observed.

### 2.5. Reaction to HSC73 Protein

**Figure 7:**
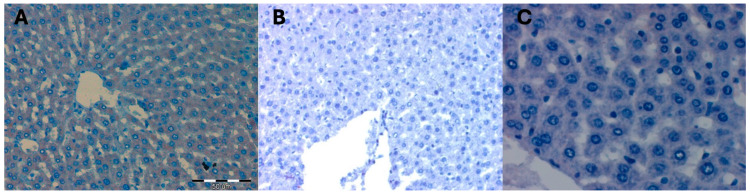
(**A**) Control group KI. Invisible immunohistochemical reaction for HSC73 in a fragment of the liver of a female rat that was decapitated on the first day of the experiment. H + AEC dyeing. (**B**) KII control group. Invisible immunohistochemical reaction for HSC73 in a fragment of the liver of a female rat that was decapitated on the 5th day of the experiment. Magnification approx. 300×. H + AEC staining. (**C**) KIII control group. Invisible immunohistochemical reaction for HSC73 in a fragment of the liver of a female rat that was decapitated on the 10th day of the experiment. Magnification approx. 500×. H + AEC staining.

No statistically significant differences were observed in the mean surface area occupied by a positive immunohistochemical reaction for the HSC73 protein in the liver of female control rats and thus not submitted to hyperbaric treatment. The color reaction for the HSC73 protein was not visible on any of the observed liver preparations (Table 10; Figure 7).

**Figure 8:**
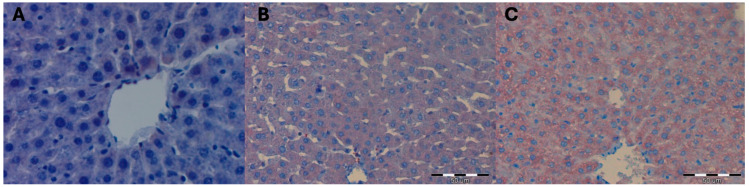
(**A**) DHI experimental group. Invisible immunohistochemical reaction for HSC73 in a fragment of the liver of a female rat that underwent hyperbaric treatment and was decapitated on the first day of the experiment, i.e., immediately after removal from the hyperbaric chamber. Magnification approx. 500×. H + AEC staining. (**B**) Experimental group DHII. Moderately severe immunohistochemical reaction for HSC73 in a fragment of the liver of a female rat that underwent hyperbaric treatment and was decapitated on the 5th day of the experiment, i.e., 5 days after removal from the hyperbaric chamber. H + AEC dyeing. (**C**) Experimental group DHIII. A strongly intensified immunohistochemical reaction for HSC73 in a fragment of the liver of a female rat, which was subjected to hyperbaric treatment and decapitated on the 10th day of the experiment, i.e., 10 days after removal from the hyperbaric chamber. H + AEC dyeing.

The mean surface area occupied by a positive immunohistochemical reaction for HSC73 in the liver of female Wistar rats subjected to high pressure and decapitated immediately after removal from the hyperbaric chamber DHI (mean 303.33 +/− 40.10) did not differ from that observed in the livers of female rats from the control groups KI (mean 350.00 +/− 26.00), KII (mean 300.67 +/− 29.00), and KIII (mean 298.00 +/− 19.29; *p* = 0.19). At 5 days after removal from the hyperbaric chamber, in the DHII group, the mean surface area occupied by a positive immunohistochemical reaction for HSC73 in the livers of female rats increased statistically significantly (mean 1009.67 +/− 327.61; *p* = 0.05) and increased statistically significantly even more after 10 days after removal from the hyperbaric chamber for the DHIII group (mean 2041.67 +/− 395.15; *p* = 0.02) (Table 11 and Table 12; Figure 8).

Hyperbaric treatment stimulated the formation of HSC73 protective proteins in the liver cells of female rats, and this process intensified over time since the animals were removed from the hyperbaric chamber, 5 days after removal, intensifying even more after 10 days after the animals were removed from the hyperbaric chamber. However, immediately after removing the animals from the hyperbaric chamber, the formation of protective proteins of the HSC73 type was not observed.

## 3. Discussion

Researchers’ growing interest in autophagy, as well as the mechanisms of its stimulation and inhibition, results from its potential possibilities in preventing and treating various diseases, including the elimination of viral genetic material. Autophagy is a specific intracellular self-cleaning process, allowing the renewal and prevention of abnormal organelle function due to wear and aging [17]. This is particularly important in liver cells, where self-cleaning plays a critical clinical role.

It is known that pressure higher than atmospheric pressure has a physical effect on the body, hindering gas exchange in the lungs and increasing respiratory muscles’ effort. It also causes damage to the wall of the alveoli and impairs sensorimotor coordination and manual skills. Biochemically, hyperbaric conditions can lead to oxygen toxicity, nitrogen narcosis, a lowered seizure threshold, and enzymatic disturbances in tissues. Therefore, hyperbaric treatment cannot remain without affecting the body’s cells [18,19]. While the effects of increased pressure on organ cells, such as liver cells, remain unexplored, our study investigated how hyperbaric conditions affect autophagy in liver cells. Previous research on hyperbaric treatment itself or with the respiratory factor of 100% oxygen on humans comprises mainly clinical trials based on the analysis of indicators in blood samples or through imaging diagnostics like X-rays, CT, or MRI.

In this study, macroautophagy was triggered in hepatocytes after exposure to 1.6 ATA for 120 min with air as a respiratory factor. Interestingly, this was a short-term effect because macroautophagy disappeared after 10 days. However, autophagy itself did not vanish: instead, it transitioned to chaperone-mediated autophagy 10 days postexposure. This suggests that hyperbaric treatment may serve as a useful tool for stimulating the purification of hepatocytes through autophagy.

The existing literature includes attempts to evaluate the effect of hyperbaric conditions on cells. The most common is the aspect of the participation of hyperbaric oxygen in processes that may slow down the aging process. For instance, Yoshinoya et al. showed that pressure of 3 ATA with a respiratory factor of 100% oxygen increases the viability and proliferation of stem cells derived from adipose tissue [20]. Similarly, studies on the effects of hyperbaric oxygen on the liver have determined that it reduces liver damage by activating endothelial vascular growth factor [21]. Additionally, Reillo MR and Altieri RJ observed antiviral effects of hyperbaric oxygen on HIV-infected cells [6]. In the face of viral infection, autophagy initiates an innate immune response, induces the production of interferon, selectively degrades immune elements associated with virus particles, and coordinates acquired immunity by presenting T cells with antigens derived from the virus. However, it turns out that some viruses can use autophagy to their advantage [14]. In the present study, the animals breathed air, and hyperbaric treatment activated autophagy, which is believed to be a powerful mechanism used by host cells to defend against viral infection. Double-membrane vesicles, called autophagosomes, deliver a trapped viral charge to the lysosome for its degradation.

An unequivocally beneficial result of increased autophagy is protein catabolism and organelle death with the subsequent use of basic components. Proteins and organelles that have any abnormalities due to defective synthesis or damaged proteins must be removed before they become toxic. Lysosomal hydrolases can degrade all types of macromolecules (proteins, lipids, nucleic acids, and complex sugars). The role of autophagy also goes far beyond the elimination of damaged cellular components and the quality control of proteins. In the liver, which is the object of interest of the current work, apart from the active division of energy stores, autophagy may contribute to the regulation of its metabolism by degrading a certain number of enzymes in various metabolic pathways. Autophagy chaperone-mediated with proteins seems to be well suited for this purpose due to its ability to target single proteins selected for degradation in lysosomes [10,22].

In this study, a statistically significant increase in chaperone-mediated autophagy was observed 5 days after the end of hyperbaric treatment, and its intensity continued to grow over time. For metabolically active organs like the liver, autophagy is essential, especially during short periods of fasting, which can stimulate maximum autophagy within just four hours [8].

Allaire et al., analyzing the process of autophagy, concluded that autophagy not only regulates hepatocyte function but also affects non-connective cells such as endothelial cells, macrophages, and liver stellate cells [23]. Autophagy dysregulation has been linked to many liver diseases, making its modulation a promising therapeutic strategy. Strengthening autophagy can prevent the progression of many liver diseases, including storage disorders, acute liver damage, nonalcoholic fatty liver disease, and chronic liver disease associated with alcoholism.

Numerous scientific studies aim to clarify the importance of autophagy dysfunction in various diseases, i.e., cancer, neurodegeneration, myopathies, infections, syndromes, and metabolic diseases. Metabolically modulated autophagy for therapeutic purposes has the potential to treat various disease states in which autophagy processes have become deregulated [24,25]. Autophagy affects the condition of the immune system in non-specific and specific immunity [7]. It has been proven that it participates in protection against viruses and bacteria [26,27,28]. However, its impact on cancer cells remains a subject of debate. It has been shown that autophagy may indicate inhibition in relation to cancer cells, but it may also affect the prolongation of their survival [29,30]. Furthermore, it has been linked to neurodegenerative diseases such as Alzheimer’s, Parkinson’s, and Huntington’s chorea, as well as inflammatory bowel diseases such as Crohn’s disease [31,32].

In recent years, researchers’ attention has been absorbed by research on autophagy in the cell nucleus, which may affect the inhibition of aging processes [33,34]. In most organs, very different factors, so-called stressors, lead to ER stress and activation of an unfolded protein response (UPR), which aims to restore homeostasis in the cell [7]. Hypoxia, oxidative stress, DNA damage, or mitochondrial dysfunction can activate macroautophagy as stressors through a wide range of signaling pathways (JNK—c-Jun N-terminal kinase, CaMKK—calcium/calmodulin protein-dependent kinase, LKB—hepatic kinase B, AKT—protein kinase B, Sirt1—sirtuin 1, PERK—protein RNA kinase-like reticulum endoplasmic kinase, PDGF—platelet-derived growth factor, AMPK—AMP-activated protein kinase) [35,36,37]. In the light of our research, it seems that hyperbaric treatment may also be included in the group of stressors that stimulate autophagy.

The stages of macroautophagy include autophagosome initiation, nucleation, elongation, fusion with the lysosome, and degradation of autophagosome content. Shortly after hyperbaric exposure, this study observed a statistically significant increase in immunohistochemical markers for autophagy proteins such as beclin 1, LC3B, and RAB7, confirming the activation of the autophagy process. These proteins are crucial for autophagosome formation and maturation, with LC3B serving as a reliable marker for monitoring autophagy activity [27,38].

In eukaryotes, autophagy initiation, charge recognition, charge absorption, and bubble closure depend on the LC3/GABARAP protein family, consisting of seven protein families (LC3A (two splicing variants), LC3B, LC3C, GABARAP, GABARAPL1, and GABARAPL2). LC3B, the most studied family protein, is associated with the development and maturation of the autophagosome and is used to monitor autophagy [39]. It is capable of connecting to the inner and outer membranes of the autophagosome [40]. There are two forms of this protein: LC3-I (LC3A), occurring in the cytoplasm, the amount of which is variable in different cell types, and LC3-II (LC3B), which is associated with the surface of autophagosomes, correlating with their number. The products of digestion taking place in the autolysosome are released back into the cytoplasm. Then, the level of LC3II inside the autolysosome decreases, and the LC3II in the cytosol is again transformed into LC3 I. A low level of LC3II in the cytoplasm, therefore, indicates a low level of autophagy. Fluorescently labeled LC3 can be observed in cells in the form of small dots, which are forming autophagosomes.

Rab proteins (Ras-related in the brain) are the largest family of small monomeric GTPs in eukaryotic cells (Ras-like GTPases). About 70 Rab proteins have been described in human cells [41,42].

In mammals, there are two Rab7 proteins: Rab7a and Rab7b. In the literature, Rab7a is referred to as Rab7 [43]. It is a key protein for autophagy. It regulates late endosome transport, participates in the formation of the autophagosome and plays an important role in the maturation of the autophagosome [41]. In mammalian cells, the Rab7 protein is located mainly within late endosomes and lysosomes, as well as phagosomes [44].

Determining the role of autophagy in physiopathological processes, regardless of the specific factors for a given disease, it is a huge challenge to examine and determine whether in a given case autophagy protects the cell or leads inevitably to its death [45]. This complexity arises because autophagy’s function can vary greatly depending on cell type and external factors, sometimes even having opposite effects [46], especially given that the disruption of autophagy at any stage of its course can lead to the development of pathological, degenerative, or disease changes.

The main breathing mixture used in diving is still air, which makes up the Earth’s atmosphere. The maximum permissible depth of diving in the air is 50 m due to nitrogen (78%), causing a narcotic effect on the brain and oxygen (21%), and leading to toxicity at increased pressure [47]. In this work, immersion to a depth of 16 m of a diver who is breathing air activated macroautophagy and increased the synthesis of chaperone proteins.

Overall, understanding autophagy’s role in physiological and pathological processes is complex, as it can have opposing effects depending on the cell type and external conditions. Despite these challenges, the ability to manipulate autophagy has enormous therapeutic potential across a broad range of diseases.

## 4. Materials and Methods

A total of 36 female Wistar rats with a body weight of 200–250 g aged 2.5–3 months were used for the study. A positive opinion was obtained for conducting tests on laboratory animals (opinion of the Second Local Ethical Committee for Animal Experiments in Lublin, Resolution No. 35/2013, 21 May 2013).

The animals were randomly divided into six equivalent groups: three control groups (KI, KII, KIII), and three experimental groups (DHI, DHII, DHIII). All animals in the experimental groups underwent hyperbaric treatment, which involved compression in a hyperbaric chamber, with a pressure of 1.6 ATA for 120 min while breathing atmospheric air. These are the most common conditions encountered by a diver underwater. This conditions also allows for breathing 100% oxygen [48].

Animals from the experimental group DHI were decapitated immediately after removal from the hyperbaric chamber, those from the DHII group were decapitated 5 days after removal, and animals from the DHIII groups were decapitated after 10 days after removal. Rats from the control groups were decapitated at the same time as the experimental groups: KI on the 1st day, KII on the 5th day and KIII on the 10th day.

After decapitation, the liver was retrieved and evaluated macroscopically. Histological preparations were also made. Liver slices taken for examination with a light microscope were fixed in 10% formalin and then dehydrated in a series of alcohols with increasing concentration (40%, 50%, 60%, 70%, 80%, 90%, 99.9%). The slices were submerged in paraffin. Paraffin blocks were cut with a rotational microtome into slices with a thickness of 3–9 μm.

Immunohistochemical reactions with beclin 1, LC3B, RAB7, and HSC73 proteins were carried out on the preparations. For immunohistochemical analysis, the three-step labeled streptavidin–biotin detection method of paraffin blocks (LSAB three-step) was used. In the first stage, the test fragment was cleaned of endogenous peroxidases by oxidation with hydrogen peroxide, and then the antigen was reacted with the primary antibody (antigen–antibody reaction). In the second stage, the primary antibody was reacted with the biotinylated secondary antibody. The third stage consisted of the reaction of biotin with avidin (ABC—avidin–biotin complex) or streptavidin (BSA—biotin–streptavidin) combined with horseradish peroxidase.

The addition of a staining material (AEC (3-amino-9-ethyl carbazole)), which was oxidized by horseradish peroxidase at the antigen–antibody junction site, produced a color reaction (red) where the antigen was sought. Photographic documentation was achieved using a color video camera (CCD-IRIS, Sony) connected to a computer.

### Statistical Analysis

The evaluation of the results of immunohistochemical tests was performed qualitatively, taking into account the intensity of the color reaction. The quantitative evaluation was performed using computer programs for morphometric analysis of microscopic images, counting the average surface areas occupied by a positive immunohistochemical reaction in individual groups for all tested antibodies. One-way analysis of variance (ANOVA) and Student’s *t*-test were used for statistical evaluation. The differences were considered statistically significant at *p* ≤ 0.05.

## 5. Conclusions

A single hyperbaric treatment at 1.6 ATA stimulated macroautophagy in liver cells, which was observed immediately after discontinuation of hyperbaric treatment. It increased after 5 days, and after 10 days dropped to the level observed in the control group.

During intensive macroautophagy, increased closure of substrates in the autophagosome (covering them with a phagophore) was observed (an increase in the activity of the beclin 1 marker). There was also increased late endosome transport and accelerated autophagosome maturation (increase in RAB7 marker activity). Furthermore, there was an increase in the number of autophagosomes (an increase in the activity of the LC3B marker) immediately after returning to atmospheric pressure, and after 5 days, it was observed that there was a statistically insignificant decrease.

Stimulation of macroautophagy once with hyperbaric treatment at 1.6 ATA weakened with the time elapsed since the cessation of high pressure, but after 5 days from the cessation of hyperbaric treatment, chaperone-mediated autophagy appeared, which intensified over time and persisted until the end of the study (increase in the amount of HSC73 chaperones in cells).

In conclusion, a one-time hyperbaric treatment (1.6 ATA) temporarily enhanced macroautophagy and promoted long-term production of HSC73 protective proteins in liver cells, making it a potential tool for stimulating short-term macroautophagy and sustained chaperone production in liver cells.

## Figures and Tables

**Table 1:** Mean surface area in μm^2^ occupied by positive immunohistochemical reaction for beclin 1 on the test surface: 781,193.35 μm^2^ in control groups. Standard deviation and one-way ANOVA. A vs. B *p* = 1; A vs. C *p* = 0.1; B vs. C *p* = 0.23.

Control Group	Liver Beclin 1			One-Way ANOVA
	KI	KII	KIII	
Mean	290.14 ^A^	309.32 ^B^	315.09 ^C^	*p* = 0.3
Standard deviation	91.05	89.12	101.2	

**Table 2:** Mean surface area in μm^2^ occupied by a positive immunohistochemical reaction for beclin 1 on the test surface: 781,193.35 μm^2^ in experimental groups. Standard deviation and one-way ANOVA. A vs. B *p* = 0.83; A vs. C *p* < 0.001; B vs. C *p* < 0.001.

Experimental Group	Hyperbaric Treatment Liver Beclin 1			One-Way ANOVA
	DHI	DHII	DHIII	
Mean	1005.35 ^A^	1100.34 ^B^	290.35 ^C^	*p* < 0.001
Standard deviation	233.21	212.45	88.2	

**Table 3:** Student’s *t*-test for statistical significance of differences between the studied control and experimental groups in the mean surface area in μm^2^ occupied by a positive immunohistochemical reaction for beclin 1.

Experimental Groups	Control Groups		
	1st Day	After 5 Days	After 10 Days
	KI	KII	KIII
DHI	*p* < 0.001		
DHII		*p* < 0.001	
DHIII			*p* = 0.9

**Table 4:** Mean surface area in μm^2^ occupied by a positive immunohistochemical reaction for LC3B on the test surface: 781,193.35 μm^2^ in the control groups. Standard deviation and one-way ANOVA. A vs. B *p* = 0.001; A vs. C *p* = 0.002; B vs. C *p* = 1.

Control Group	Liver LC3B			One-Way ANOVA
	KI	KII	KIII	
Mean	302.33 ^A^	350.00 ^B^	350.00 ^C^	*p* = 0.0004
Standard deviation	8.74	6.10	8.04	

**Table 5:** Mean surface area in μm^2^ occupied by a positive immunohistochemical reaction for LC3B on the test surface: 781,193.35 μm^2^ in the experimental groups. Standard deviation and one-way ANOVA. A vs. B *p* = 0.99; A vs. C *p* = 0.01; B vs. C *p* = 0.01.

Experimental Group	Hyperbaric Treatment Liver LC3B			One-Way ANOVA
	DHI	DHII	DHIII	
Mean	1042.67 ^A^	1038.33 ^B^	305.00 ^C^	*p* = 0.0003
Standard deviation	367.86	294.38	42.72	

**Table 6:** Student’s *t*-test for the statistical significance of differences between the studied control and experimental groups in the mean surface area in μm^2^ occupied by a positive immunohistochemical reaction for LC3B.

Experimental Groups	Control Groups		
	1st Day	After 5 Days	After 10 Days
	KI	KII	KIII
DHI	*p* < 0.001		
DHII		*p* < 0.001	
DHIII			*p* = 0.93

**Table 7:** Mean surface area in μm^2^ occupied by a positive immunohistochemical reaction for RAB7 on the test surface: 781,193.35 μm^2^ in control groups. Standard deviation and one-way ANOVA. A vs. B *p* = 0.77; A vs. C *p* = 0.96; B vs. C *p* = 0.71.

Control Group	Liver RAB7			One-Way ANOVA
	KI	KII	KIII	
Mean	293.00 ^A^	304.00 ^B^	292.00 ^C^	*p* = 0.90
Standard deviation	33.60	50.12	13.11	

**Table 8:** Mean surface area in μm^2^ occupied by a positive immunohistochemical reaction for RAB7 on the test surface: 781,193.35 μm^2^ in the experimental groups. Standard deviation and one-way ANOVA. A vs. B *p* = 0.83; A vs. C *p* = 0.001; B vs. C *p* = 0.001.

Experimental Group	Hyperbaric Treatment Liver RAB7			One Way ANOVA
	DHI	DHII	DHIII	
Mean	1006.67 ^A^	1030.00 ^B^	296.66 ^C^	*p* < 0.005
Standard deviation	90.18	157.16	40.41	

**Table 9:** Student’s *t*-test for the statistical significance of differences between the studied control and experimental groups in the mean surface area in μm^2^ occupied by a positive immunohistochemical reaction for RAB7.

Experimental Group	Control Group		
	1st Day	After 5 Days	After 10 Days
	KI	KII	KIII
DHI	*p* = 0.001		
DHII		*p* = 0.02	
DHIII			*p* = 0.87

**Table 10:** Mean surface area in μm^2^ occupied by a positive immunohistochemical reaction for HSC73 on the test surface: 781,193.35 μm2 in control groups. Standard deviation and one-way ANOVA. A vs. B *p* = 0.09; A vs. C *p* = 0.06; B vs. C *p* = 0.91.

Control Group	Liver HSC73			One-Way ANOVA
	KI	KII	KIII	
Mean	350.00 ^A^	300.67 ^B^	298.00 ^C^	*p* = 0.07
Standard deviation	26.00	29.00	19.29	

**Table 11:** Mean surface area in μm^2^ occupied by a positive immunohistochemical reaction for HSC73 on the test surface: 781,193.35 μm^2^ in experimental groups. Standard deviation and one-way ANOVA. A vs. B *p* = 0.05; A vs. C *p* = 0.02; B vs. C *p* = 0.02.

Experimental Group	Hyperbaric Treatment Liver HSC73			One Way ANOVA
	DHI	DHII	DHIII	
Mean	303.33 ^A^	1009.67 ^B^	2041.67 ^C^	*p* < 0.001
Standard deviation	40.10	327.61	395.15	

**Table 12:** Student’s *t*-test for the statistical significance of differences between the studied control and experimental groups in the mean surface area in μm^2^ occupied by a positive immunohistochemical reaction for HSC73.

Experimental Groups	Control Groups		
	1st Day	After 5 Days	After 10 Days
	KI	KII	KIII
DHI	*p* = 0.19		
DHII		*p* = 0.05	
DHIII			*p* = 0.02

## Data Availability

The datasets generated during and/or analyzed during the current study are not publicly available, but are available from the corresponding author on reasonable request.
